# Magnitude of neonatal sepsis and its associated factors among neonates admitted to the neonatal intensive care unit in Hawassa Comprehensive Specialized Hospital, Sidama Regional State, Ethiopia, 2020

**DOI:** 10.1186/s12884-024-06583-z

**Published:** 2024-05-22

**Authors:** Frehiwot Sisay Baheru, Bisrat Zeleke Shiferaw, Tigistu Toru, Fisha Alebel GebreEyesus

**Affiliations:** 1 Hawassa Comprehensive Specialized Hospital, Midwifery Metron, Hawassa, Ethiopia; 2 Department of Nursing, College of Medicine and Health Sciences, Wolkite University, PO Box 07, Wolkite, Ethiopia; 3 Department of Nursing, College of Medicine and Health Sciences, Wolayita Sodo University, Wolayita, Ethiopia

**Keywords:** Sepsis, Neonates, Neonatal intensive care unit, Magnitude, Hawassa, Ethiopia

## Abstract

**Background:**

Neonatal sepsis is one of the most common causes of disease and death among neonates globally. And it made a great contribution to neonatal admission to intensive care units. To mitigate the ongoing neonatal crisis and accomplish the goal of sustainable development through a decrease in neonatal mortality, information from various regions is needed. Despite the considerable burden of neonatal sepsis in our setting, no prior studies were conducted in the study area. So, this study aimed to assess the magnitude and associated factors of neonatal sepsis among neonates admitted to the neonatal intensive care unit at Hawassa University Comprehensive Specialized Hospital, Sidama Regional State, Ethiopia.

**Methods:**

A hospital-based cross-sectional study was carried out among 287 neonates from March 1, 2020, to April 25, 2020. An interviewer-administered structured questionnaire was used to collect the data. The data were cleaned, coded, and entered into Epi Data 3.1 software and exported to Statistical Package for Social Science (SPSS) software version 23.0 for analysis. Binary logistic regression analyses were performed to identify variables having a significant association with neonatal sepsis. A p-value of ≤ 0.05 was considered statistically significant during multivariable logistic regression.

**Results:**

The study found that the magnitude of neonatal sepsis was 56%. The mean age of neonates was 3.2(SD±2.2) days. Around two-fifths (39%) of neonates were in the gestational age of <37 completed weeks. A quarter of mothers(25.8%) were delivered through cesarean section. During labor, 251 (87.5%) mothers had ≤4 digital vaginal examinations. Moreover, the finding revealed that mothers who delivered by cesarean section [AOR = 2.13, 95% CI (1.090-4.163)]. neonates who had been resuscitated at birth [AOR = 4.5, 95% CI (2.083-9.707)], and neonates who had NG tube inserted [AOR = 4.29, 95% CI (2.302-8.004)] were found to be significantly associated with neonatal sepsis.

**Conclusions:**

The current study shows that neonatal sepsis was prevalent among more than half of the neonates admitted to the NICU. Therefore, designing strategies to enhance the aseptic techniques of professionals in the provision of care and actively and collaboratively working with cluster health facilities is highly recommended.

## Background

Neonatal sepsis is a systemic inflammatory response syndrome in the presence of infection during the first 28 days of life and includes septicemia, pneumonia, meningitis, arthritis, osteomyelitis, and a urinary tract infection [1, 2]. Based on the onset of symptoms, neonatal sepsis could be divided into early-onset neonatal sepsis (EOS) and late-onset neonatal sepsis (LOS). Early-onset sepsis occurs within the first 72 hours of life and is transmitted both prenatally and intrapartum, and late-onset sepsis occurs after 72 hours of childbirth and is primarily acquired horizontally from the environment [2–4].

The World Health Organization 2021 report estimated between 1.3 million and 3.9 million cases of neonatal sepsis annually, leading to 400,000–700,000 deaths [5]. It was reported that sub-Saharan Africa, South Asia, and Latin America had the highest burden of neonatal sepsis with a case fatality risk of 9.8% in the first month of life [2, 6]. Compared to children born in high-income countries, those born in sub-Saharan Africa had a 10-fold higher risk of dying in the first month of life [7].

Neonatal sepsis has an economic impact that results from increased medical costs, prolonged hospital stays, and potentially poor long-term neurodevelopmental outcomes [8]. Neonatal sepsis is responsible for 1.6 times the global number of childhood deaths as malaria, and over four times the number of childhood deaths caused by HIV [6]. Despite its disproportionate burden on childhood morbidity and mortality, neonatal sepsis receives substantially less investment internationally as a public health priority compared with other major conditions [9]. In Ethiopia, neonatal mortality is unacceptably high at 30 per 1,000 live births, which is far from the global Sustainable Development Goal (SDG) target of reducing neonatal mortality to 12 per 1,000 live births by 2030 [10–12]. To reduce this huge amount of neonatal mortality, several initiatives were carried out, but the reduction in neonatal death rate does not appear to be leading to the desired outcome. These interventions include clean umbilical cord cutting, early detection and treatment of infectious disease, intrapartum antibiotic prophylaxis for mothers who are at risk, and improvements in perinatal care services to prevent neonatal sepsis [13–16].

Ethiopia's neonatal mortality rates are primarily caused by preterm (37%) followed by infection (28%), and asphyxia(24%) [8]. As documented in various works of a research article, neonatal sepsis is caused by factors related to both maternal and neonatal conditions. Birth weight, prematurity, birth asphyxia [17–21], resuscitation at birth, and surgery were significant predictors of [17, 22, 23] neonatal sepsis. Maternal factors such vaginal colonization with group B streptococcus (GBS), A history of a previous infant with GBS infection, duration of labor, mode of delivery, and place delivery [6, 18, 24] were associated with neonatal sepsis.

Numerous studies conducted in Ethiopia revealed that the magnitude of neonatal sepsis varied greatly depending on the geographic location [4, 16–21, 25–31] ranging from 11.7% in North-West Ethiopia to 78.3% in South Ethiopia and 79.4% in North-East Ethiopia [4, 20, 32]. Considering that Sidama Regional State is one of Ethiopia's newest regional states, no previous research has been done despite the high rate of newborns admitted to the neonatal intensive care unit (NICU) with infections, most commonly neonatal sepsis, recognizing that various malfeasances exist in the area that could predispose newborns to infections, as well as the result of numerous studies carried out nationally that have demonstrated the magnitude of neonatal sepsis with great inconsistencies across different geographical regions. Regular evaluations of the issue at each site are essential. Therefore, determining the extent and contributing causes to neonatal sepsis in a particular community will be essential to preventing and reducing the associated burden of neonatal disease and mortality, which will ultimately help to improve neonatal health overall. Thus, the main objective of this study is to assess the magnitude and associated factors of neonatal sepsis among neonates admitted to the Neonatal Intensive Care Unit (NICU) at Hawassa University Comprehensive Specialized Hospital (HUCSH), Sidama Regional State, Ethiopia, in 2020.

### Methods

#### Study design

A hospital-based cross-sectional study was carried out.

#### Study area and period

The study was carried out at Hawassa Comprehensive Specialized Hospital. The hospital is located at Hawassa City, the capital of Southern Nations Nationalities and People's Region, which is 275 km far from the capital city of the country, Addis Ababa. This hospital was established in November 2005 and it is the largest hospital in the region; it serves as a teaching, training, and clinical service center. It has about 400 beds and provides health care services for more than 18 million people in the Southern regions of Ethiopia, and two zones of the Oromia region. The Neonatology unit of the hospital has 9 pediatricians and 25 Nurses. It is well-equipped and gives a full range of Neonatal services. Total Neonate admissions in Hawassa Compressive Specialized Hospital was 1, 700 per year in 2019. The study was carried out from March 1, 2020, to April 25, 2020.

### Population

#### Source population

All neonates who were admitted and treated at the NICU at Hawassa University Comprehensive Specialized Hospital

#### Study population

Sampled neonates who were admitted to the neonatal intensive care unit in Hawassa University Comprehensive Specialized Hospital.

### Inclusion and exclusion criteria

#### Inclusion criteria

All neonates who were admitted to Hawassa University Comprehensive Specialized Hospital at the neonatal intensive care unit.

#### Exclusion criteria

All neonates whose mothers died of birth and without family who could not disclose their information, mothers who had hearing impairments or were unable to talk. neonates who were discharged early before data collection, neonates whose cards had incomplete information, and who died on arrival were excluded from the study.

### Sample size determination

The minimum sample size required for the study was determined by using the single population proportion formula by taking the magnitude of neonatal sepsis 21 % from a study conducted at Shashemenne town [26].

$$\textstyle\frac{\mathrm N=\mathrm Z^2\;\mathrm{pq}}{\mathrm d^2}$$,

where:

$$\begin{array}{lc}\mathrm d=\;\mathrm{margin}\;\mathrm{of}\;\mathrm{error}\;(5\%\;=\;0.05)&\\\begin{array}{lc}\mathrm p=\;\mathrm{margin}\;\mathrm{estimate}\;\mathrm{of}\;\mathrm{population}\;\mathrm{proportion}\;(21.0\%\;=\;0.21)\end{array}&\\\mathrm q\;=\;1-\mathrm P\;=\;0.79&\\\mathrm Z\;=\;\mathrm{confidence}\;\mathrm{level}\;(95\%\;=\;1.96)&\end{array}$$ 

The sample size was:

$$\frac{\mathrm{ni}=\mathrm z^2\;\mathrm{pq}}{\mathrm d^2}=\frac{\left(1.96\right)^2\;\left(0.779\right)\;\left(0.221\right)}{\left(0.05\right)^2}=261$$

By considering a 10% non-response rate, the final minimum adequate sample size was 287

#### Sampling technique and procedure

The study population consisted of neonates who were admitted and treated in NICUs during the study period. A systematic random sampling procedure was used to select study participants from the list of admitted newborns at the NICU. The total number of neonates (N) was estimated by considering the client flow in the last year by reviewing the registration of neonates, which yielded a total of 1700 neonates. Then the number of sampling intervals was determined by dividing the number of neonates by the estimated sample size, and hence the sampling interval (k) was determined. The first sampling unit was selected by the lottery method, and every other neonate admitted with sepsis was taken until it reached the sample size.

### Variables

#### Dependent variable

Neonatal sepsis (Yes/No)

#### Independent variables

Socio-demographic characteristics: Age of neonate, sex of neonate, maternal age, residence, educational status of the mother. and monthly income

Maternal factors: Parity, history of Urinary Tract Infection (UTI), foul-smelling amniotic fluid, meconium-stained amniotic fluid, Antenatal care (ANC), duration of rupture of membrane, the person assisting delivery, place of delivery, frequency of pervaginal (PV) examination, maternal fever

Neonatal factors: Prematurity, low birth, Apgar score status, breastfeeding ability, birth defect/anomaly, surgical procedure, resuscitation, intubation, umbilical catheterization.

### Operational definition

#### Neonatal *sepsis*

Neonates with the presence of at least one clinical sign plus at least two laboratory results that are suggestive of neonatal sepsis or neonates who are diagnosed as sepsis by an attending physician and fulfill sepsis criteria within 0-28 days of life [30]. Along with the established Integrated Management of Neonatal and Childhood Illness (IMNCI) clinical features, including the presence of two or more persistent fever (≥37.5 °C) or persistent hypothermia (≤35.5 °C) for more than one hour, fast breathing (≥60 breath per minute), severe chest in drawing, grunting, not feeding well, movement only when stimulated, bulged fontanel, convulsion, lethargic or unconsciousness along with ≥2 of the hematological criteria such as total leukocyte count (< 4000 or > 12,000 cells/mm3), absolute neutrophil count (< 1500 cells/mm3 or > 7500 cells/mm3), platelet count (< 150 or > 450 cells/mm3), and random blood sugar (< 40 mg/dl or > 125 mg/dl) were used to diagnosing neonatal sepsis [16, 28, 33, 34].

Early-onset neonatal sepsis is defined as sepsis that occurs within 72 hours of life [2, 3, 34, 35].

Late-onset sepsis is defined as sepsis that occurs after 72 hours of life [2, 3, 34, 35].

#### Data collection instrument and procedure

Data were collected using structured, interviewer-administered, and pre-tested questionnaires which contain three main parts; socio-demographic characteristics of the child and caregiver, maternal-health related, and neonatal-health related factors through face-to-face interviews of the caretaker. A chart review was also done to identify the neonatal reasons for admission and lab investigation was taken from the client chart. Data were collected by four midwives who are fluent in the local language’ and currently working in Hawassa University’s comprehensive specialized hospital. Close supervision of trained data collectors was undertaken by the trained supervisors (two BSc nurses). The supervisor strictly supervised the data collection process and provided on-site advice and feedback to the data collectors daily. The principal investigator examined the completeness, consistency, and accuracy of the collected data regularly.

#### Data quality control

The quality of data was ensured through the training of data collectors and pretesting of the questionnaire. The questionnaires were adapted from previously published research articles and other related literature [16, 18–22, 25, 26, 36]. Then the adapted questionnaires were modified and conceptualized to fit the research objectives, and data was gathered by a healthcare worker who had a better experience in data collection. The questionnaire was translated into Amharic by an expert translator and then back to English to ensure the consistency of questions (meaning). The questionnaire was pre-tested on 5% (15 neonates with caregivers) living in Shashemenie town. In addition to this, the instrument was tested for reliability and validity, and accordingly; Cronbach's alpha coefficient was found to be 0.784. Then, necessary corrections were made to the tool before actual data collection.

#### Data processing and analysis

The data were coded, cleared, and entered into Epi data 3.1 software and exported to Statistical Package for Social Science (SPSS) software version 23.0 for analysis.

Categorical variables were summarized into percentages and proportions. The continuous variables were summarized into means, medians, standard deviation, and ranges, and the results were presented with tables and figures. Both bivariate and multivariable analyses were used to see the association of different variables. Those variables that revealed a statistically significant value at a p-value of $\le$ 0.25 in the bivariable analysis were selected for multivariable logistic regression. For model fit, the Hosmer and Lemeshow test was carried out and found to be (0.738) which indicated the final model was well fitted and The multicollinearity effect among candidate variables was carried out (checked) using variance inflation factor (VIF) and tolerance test between independent variables and found to be VIF < 5(1.03-4.40) and tolerance test >0.2.

An adjusted odds ratio with a 95% confidence interval was used to measure the degree of association between variables. A *P*-value of < 0.05 was considered statistically significant during multivariable logistic regression.

#### Ethical considerations

The ethical clearance of the study was obtained from the Institutional Review Board of Pharma College of Health Science with reference numbers PMC/ECL/009/2020 Then an official letter was submitted to the hospital medical director's office and data collection was performed after obtaining permission. the purpose of the study and its procedures were explained to the mothers of the newborn. Prior to data collection, informed verbal and witten consent was obtained from the parents and/or guardians of the newborn. The informed consent was acceptable and approved by the ethical review committee. The respondents’ rights to refuse or withdraw from participating in the study were also fully acknowledged. All information obtained throughout the study was kept confidential. This study was conducted per the Declaration of Helsinki.

## Results

### Socio-demographic and economic characteristics of mothers

A total of 287 neonates who were admitted to NICU with their mothers were included in this study with an overall response rate of 100%. Based on this study, the mean age of mothers was 26.9 Standard deviation (SD±4.6) years. Most of the mothers of the neonates were from urban areas (59.9%) and most of them 256(88.2%) were married (Table 1).

**Table 1:** Socio-demographic and economic characteristics of mothers with their index neonates, Hawassa city administration, Sidama Regional State, Ethiopia, 2020

**Variables**	**Category**	**Frequency (*N* = 287)**	**Percent**
**Mother’s age in Years**	≤20	30	10.5
	21-34	241	84.0
	≥35	16	5.5
**Marital Status**	Single	22	7.7
	Married	253	88.2
	Widow	9	3.1
	Divorced	3	1.0
**Maternal Education**	Cannot read and write	108	37.6
	Read and write but no formal education	22	7.7
	Primary	37	12.9
	Secondary	50	17.4
	College and higher	70	24.4
**Religious Status**	Orthodox	102	35.5
	Muslim	95	33.1
	Catholic	8	2.8
	Protestant	82	28.6
**Occupational Status of mother**	Housewife	122	42.5
	Civil servants	45	15.7
	Businesswoman	62	21.6
	Private organization	15	5.2
	Daily laborer	26	9.1
	Student	17	5.9
**Residence**	Urban	169	58.9
	Rural	118	41.1
**Monthly household income**	< 2000 ETB	66	23.0
	2001 to 4000 ETB	86	30.0
	> 4000 ETB	135	47.0

*ETB* Ethiopian Birr

### Maternal-related factors for neonatal *sepsis*

Of the total mothers of the neonates, more than half 164 (57.1%) were primiparous 234 (81.5%) had antenatal care visits for the index neonate, of which 105 (36.6%), and 79 (27.5%) of them had three and four visits respectively. Concerning the place of delivery, all mothers gave birth in health facilities, 191 (66.6%) and 96 (33.4%) delivered in hospitals and health centers respectively. and three-fifths of 174 (60.6%) mothers gave birth with spontaneous vaginal delivery followed by 74 (25.8) with cesarean section. During labor, 251 (87.5%) mothers had ≤4 digital vaginal examinations, 200 (69.7%) mothers had brown (green) discoloration of amniotic fluid, and 80 (27.9) had foul-smelling amniotic fluid. Besides, 81(28.2%) mothers had a history of pregnancy-induced hypertension (Table 2).

**Table 2:** Maternal health-related factors in Hawassa University Comprehensive Specialized Hospital in Hawassa City administration, Sidama Regional State, Ethiopia, 2020

**Variables**	**Category**	**Frequency (*N* = 287)**	**Percent (%)**
**Mother’s parity**	Primiparous	164	57.1
	Multiparous	123	42.9
**ANC visit for the index neonate**	Yes	234	81.5
	No	53	18.5
**If yes, the frequency of ANC visit**	One	8	2.8
	Two	41	14.3
	Three	105	36.6
	Four	79	27.5
	More than four	2	0.7
**Place of delivery**	Hospital	191	66.6
	Health center	96	33.4
**Mode of delivery (If the place of delivery was a hospital/health center)**	Cesarean section	74	25.8
	Instrumental vaginal delivery	39	13.6
	Spontaneous vaginal delivery	174	60.6
**Who helped you during delivery?**	Health professional	286	99.7
	Other	1	0.3
**Duration of rupture of membrane**	Less than 1 hour	2	0.7
	1-6 hour	103	35.9
	More 6 hour	40	13.9
	Do not know	142	49.5
**Was the amniotic fluid brown/green discoloration**	Yes	200	69.7
	No	87	30.3
**How many times did the birth attendant perform a vaginal examination?**	<=4	251	87.5
	>4	36	12.5
**Maternal fever during this labor**	Yes	186	64.8
	No	101	35.2
**Was the amniotic fluid foul-smelling?**	Yes	80	27.9
	No	207	72.1
**Pregnancy-related hypertension/ Eclampsia of neonate**	Yes	81	28.2
	No	206	71.8
**Any bleeding during the pregnancy of this neonate/APH**	Yes	46	16.0
	No	241	84.0
**Maternal UTI/STI during the pregnancy of this neonate**	Yes	18	6.3
	No	269	93.7

*ANC* Ante Natal Care, *UTI* Urinary Tract Infection, *APH* Ante Partum Hemorrhage, *STI* Sexually Transmitted Infection

### Neonatal-Related factors for neonatal *sepsis*

Based on this study, the mean age of neonates was 3.2(SD±2.2) days and more than half 157(54.7%) of them were females. Concerning gestational age, about three-fifths (58.9%) and two-fifths (39%) of neonates were in the gestational age of 38-42 and <37 completed weeks respectively. About 286(99.7%) and 250(87.1%) neonates had an Apgar score of ≤7 in the first and fifth minutes respectively. about half of neonates 147(51.2%) had low birth weight (<2500grams). About two-thirds of 194(67.6%) and three-fourths of 215(74.9%) neonates did not cry and had been resuscitated at birth respectively. Also, 234(81.5%) of neonates were on oxygen, and 128(44.6%), 90(31.4%), and 16(5.6%) of them were administered oxygen through a nasal cannula, mask, and intranasal catheter respectively. Moreover, about half of neonates 145(50.5%) had an NG tube inserted (Table 3).

**Table 3:** Neonatal health-related factors in Hawassa University Comprehensive Specialized Hospital in Hawassa City administration, Sidama Regional State, Ethiopia, 2020

**Variables**	**Category**	**Frequency (*N* = 287)**	**Percent (%)**
**Neonate’s age in days**	≤7days	272	94.8
	>7days	15	5.2
**Neonate’s sex**	Female	157	54.7
	Male	130	45.3
**Gestational age in weeks**	<37	112	39.0
	38 to 42	169	58.9
	>42	6	2.1
**APGAR score at 1**^**st**^** minute**	≤7	286	99.7
	>7	1	0.3
**APGAR score at 5**^**th**^** minute**	≤7	250	87.1
	>7	37	12.9
**Birth weight in grams**	<2500	147	51.2
	2500-4000	123	42.9
	≥4000	17	5.9
**The neonate cried immediately after birth**	Yes	93	32.4
	No	194	67.6
**Neonate resuscitated at birth**	Yes	215	74.9
	No	72	25.1
**Neonates had any type of surgery**	No	287	100.0
**Neonate was on oxygen**	Yes	234	81.5
	No	53	18.5
**If yes, the mode of oxygen administration**	Intranasal catheter	16	5.6
	Mask	90	31.4
	Nasal cannula	128	44.6
**Neonate had endotracheal intubation**	Yes	145	50.5
	No	142	49.5
**NG tube inserted for neonate**	Yes	145	50.5
	No	142	49.5
**Umbilical catheter inserted for neonate**	Yes	2	0.7
	No	285	99.3
**Urinary catheter inserted for neonate**	Yes	2	0.7
	No	285	99.3

### Prevalence of main clinical signs of neonatal *sepsis*

Accordingly, the main clinical signs to diagnose neonatal sepsis as recommended by WHO, out of 287 neonates admitted to NICU, more than two-thirds 197(68.6%) had respiratory rate>60breaths/min; among them, 141(49.1%) had developed neonatal sepsis and about 109(38%) had temperature instability (>37.5oC or <35.5oC); out of this, 92(32.1%) had developed neonatal sepsis. About 51(17.8%) of neonates had severe chest indrawing and 48(16.7%) of them developed neonatal sepsis. Besides, 117(40.8%) of neonates had grunting; among them, 105(36.6%) were suffering from neonatal sepsis; and 45(15%) of neonates had difficulty feeding; out of the 42(14.6%) developed neonatal sepsis.

### The magnitude of neonatal *sepsis*

**Fig. 1:**
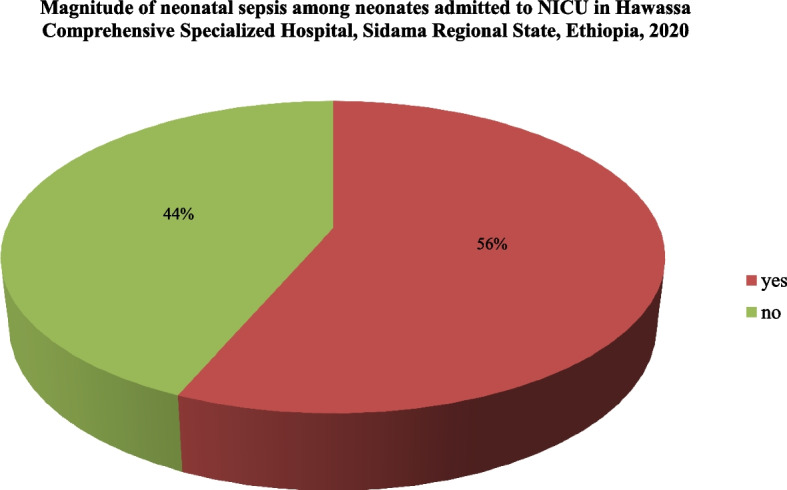
Magnitude of neonatal sepsis among neonates admitted to the neonatal intensive care unit in Hawassa Comprehensive Specialized Hospital, Sidama Regional State, Ethiopia, 2020 (*N*= 287)

Among 287 neonates admitted to NICU, 162(56%) had neonatal sepsis whereas 125(44%) had been admitted for other diseases (Figure 1).

### Factors associated with neonatal *sepsis*

To assess the association of different independent variables with neonatal sepsis, bivariable logistic regression analysis was conducted, and for a crude association, all variables with a *p*-value less than 0.25(*P*-Value<0.25) became candidates for multivariable logistic regression. Among the candidate variables, mode of delivery, ANC follow-up, maternal fever, brown (green) discoloration of amniotic fluid, birth weight, Apgar score at 5^th^ minute, with an immediate cry of the neonate at birth, resuscitation at birth, and neonate with NG tube inserted were significantly associated with the outcome variable.

From a multivariable analysis, mode of delivery, resuscitation at birth, and neonate with NG tube inserted were found to be significantly associated with the outcome variable at *P*-value < 0.05.

This study showed that mothers who delivered by Caesarean section were about two folds more likely to have their neonate's higher chance of developing neonatal sepsis than those delivered by spontaneous vaginal delivery [AOR = 2.13, 95% CI (1.090-4.163)]. Similarly, the analysis revealed that neonates who had been resuscitated at birth were 4.5 times more likely to develop sepsis than those without resuscitation at birth [AOR = 4.5, 95% CI (2.083-9.707)]. Our study also found that neonates who were NG tube inserted were at>4 times the high risk for developing neonatal sepsis compared to those without NG tube [AOR = 4.29, 95% CI (2.302-8.004)] (Table 4).

**Table 4:** Association between Predictor variables and Neonatal sepsis in Hawassa University Comprehensive Specialized Hospital ICU, Hawassa City administration, Sidama Regional State, Ethiopia, 2020 (*N*= 287)

**Variable**		**Neonatal sepsis**		**COR (95% CI)**	**AOR (95% CI)**
		No (%)	Yes (%)		
**Mode of delivery (at Hospital/health center)**	SVD	81(64.8)	93(57.4)	1	
	IVD	19(15.2)	20(12.3)	1.86(1.044-4.108)**	
	Cesarean section	25(20)	49(30.2)	1.71(1.097-3.008)**	2.13(1.09-4.16)***
**ANC follow up**	No	30(24)	23(14.2)	1	
	Yes	95(76)	139(85.8)	1.91(1.045-3.487)**	
**Maternal Fever**	No	55(44)	46(28.4)	1	
	Yes	70(56)	116(71.6)	1.98(1.212-3.238)**	
**Amniotic fluid (brown/green)**	No	51(40.8)	36(22.2)	1	
	Yes	74(59.2)	156(96.3)	2.41(1.442-4.034)**	
**Neonatal age in days**	>7	9(7.2)	6(3.7)	1	
	≤7	116(92.8)	156(96.3)	2.02(0.698-5.826)*	
**Neonatal birth weight in gram**	≥4000	13(10.4)	4(2.5)	1	
	2500-4000	67(53.6)	56(34.5)	2.71(1.647-4.467)*	
	<2500	45(36)	102(63)	7.37(2.277-23.837)**	
**Apgar scored in the 5**^**th**^** minute**	>7	32(25.6)	5(3.1)	1	
	≤7	93(74.4)	157(96.9)	10.8(4.068-28.695)**	
**Immediately cry after birth**	No	58(46.4)	136(84)	1	
	Yes	64(53.6)	26(16)	0.17(0.096-0.286)**	
**Resuscitation at birth**	No	59(47.2)	13(8)	1	
	Yes	66(52.8)	149(92)	10.25(5.26-19.96)**	4.5(2.083-9.707)***
**Neonate was on oxygen**	No	45(36)	8(4.9)	1	
	Yes	80(64)	154(95.1)	10.83(4.87-24.08)**	
**Neonate had NG tube inserted**	No	95(76)	47(29)	1	
	Yes	30(24)	115(71)	7.75(4.549-13.197)**	4.29(2.30-8.004)***

*SVD* Spontaneous vaginal delivery, *IVD* Instrumental vaginal delivery, *NG* Nasogastric

(*) refers to candidate variables for multivariable analysis at *P*-Value between 0.05 and 0.25 and 1 shows the reference group

(**) refers to candidate variables for multivariable analysis and statistically significant in bivariable logistic regression at *P*-value <0.05

(***) refers to variables significant in multivariable analysis and predictors for neonatal sepsis at *P*-value <0.05

## Discussion

The results of this study demonstrated that the risk of neonatal sepsis was significantly influenced by both maternal and neonatal-related factors. Neonatal and maternal characteristics that were substantially linked with neonatal sepsis in the current study included the mode of delivery (Cesarean section), resuscitation at birth, and having an NG tube inserted.

The magnitude of neonatal sepsis in the present study was found to be 56%. This finding is relatively congruent to the study finding in Gondar (59%) [21], Jimma(52.6%) [25], and Iran (51.8%) [37] and it is lower than the study finding in Arbaminch (78.3%) [20], Shashemene (77.9%) [26], and Sudan (65.8%) [38]. But the present study finding was relatively higher than the study finding in Gondar (11.1%) [32], Wolayita sodo(33.85%) [39], Oromiya, Arsi (34%) [19]**,** Tanzania(31.4%,49.8%) [36, 40], systemic review and meta-analysis in developing country (29.2%) [41], India (19%) [42], Jordan (12%) [43], Taiwan(15.2%) [44] and Srilanka (4.6%) [45]. The reason for this variation might be due to variations in Socio-demographic characteristics, sample size, study design, accessibility of health facility, skilled manpower/personnel, advanced equipment/health system, and differences in the way neonatal sepsis has been asserted as well as the techniques used to operationally define neonatal sepsis.

According to our research, neonatal sepsis occurred in 48.4% of babies born to mothers who had ANC visits. This finding is higher than the studies conducted in Northwest parts of Ethiopia in which 30.1% and 24.7% of neonates developed sepsis [17, 46]. Variations in the depth and nature of counseling provided to the woman during ANC, the attention paid by hospital managers, and the degree of experience and training that health professionals have acquired could all be contributing factors to the potential discrepancy.

Another maternal-related factor that raises the risk of neonatal sepsis is meconium-stained amniotic fluid (MSAF). Babies born to mothers who have MSAF are more likely to aspirate, which can constrict the lung's smaller airways and air sacs. This can encourage the growth of bacteria, ultimately leading to sepsis. According to the current study, sepsis affected approximately 44% of newborns born to moms with MSAF. This finding is relatively higher than the study finding from the Northwest part of Ethiopia (18.6%) and Shashemene town, Oromia region (4.1%) [17, 26]. Potential explanations for this variation include the majority of mothers delivered in the study area having preeclampsia or hypertension, infections, being delivered at a late gestational age, and the majority of neonates having fetal distress, which could be the cause of this discrepancy.

One of the neonatal-related factors that increases the risk of neonatal sepsis is gestational age. In the current investigation, sepsis was found in 28.2% of newborns with gestational ages under 37 and over 37 full weeks. This finding is relatively lower than the study findings in Arbaminch General Hospital, Southern Ethiopia, and Shashemene, Oromia region [20, 21]. But is relatively higher than the studies conducted in northwest parts of Ethiopia [46], Mekelle (North Ethiopia) [47], and Ghana [22]. Despite the undeveloped immunity of neonates, particularly those born before 37 weeks of gestation, this variation may be caused by intrauterine factors, factors associated with the health facility, and different procedures during and after delivery.

This study revealed that cesarean-section delivery was statistically associated with the probability of causing neonatal sepsis in which case newborns delivered through cesarean-section were two folds more likely to have a higher chance of developing neonatal sepsis than those delivered by spontaneous vaginal delivery. This finding is consistent with the study findings in Ghana [22] and Iran [48]. Vaginal and fecal bacteria exposure is rare in newborns delivered by cesarean section; however, in this instance, the development of neonatal sepsis may be linked to delayed breastfeeding initiation, inadequate aseptic technique during the procedure, absence of room fumigation, an extended second stage of labor, and an extended hospital stay. Furthermore, delaying the start of nursing after CS may prevent the neonate from benefiting from colostrum's immunity-boosting properties and its ability to guard against many pathogenic microorganisms that could otherwise negatively impact the newborn's survival.

Our research revealed a substantial association between newborn sepsis and resuscitation at birth. Compared to babies who were not given resuscitation at delivery, those who did were almost five times more likely to experience sepsis Our finding investigated that resuscitation at birth was significantly associated with neonatal sepsis. Neonates who had been resuscitated at birth were nearly five times more likely to develop sepsis than those without resuscitation at birth. This finding is congruent with findings from other studies: West Shewa zone, central Ethiopia [28], North East Ethiopia [4], Northwest parts of Ethiopia [17, 46], Jimma [25], Ghana [49], Tanzania [36, 40] and Bangladesh [50]. This might be related to contaminated and forceful procedures during resuscitation that might cause laceration and easy breakage of the mucous membrane of the newborn which forms a better way for entering microorganisms from unsterile materials. In addition to this, the available information suggests that resuscitation of neonates poses a risk of sepsis for patients with weakened immune systems, such as elderly patients, infants, and hospitalized patients. Ineffective resuscitation techniques and a disregard for recommendations by medical staff may put the newborn at higher risk of sepsis, and this finding may hold for the current study as well. Furthermore, the newborn's respiratory secretions are copious and the lumen of their peripheral airway is narrow, both of which may put them at risk for atelectasis. In addition to causing bruises to the newborn's delicate and fragile mucous membrane, carrying out strenuous procedures may cause the lungs to collapse and provide a pathway for microbes to enter the body.

The current study also revealed that nasogastric tube insertion (NGT) was a neonatal-related factor that was significantly associated with neonatal sepsis. Neonates who were NG tube inserted were >4 times the high risk for developing sepsis compared to those without NG tube This finding was relatively in line with study findings from Northwest Ethiopia [17], Turkey [37], and Taiwan [44]. It might be the result of not performing procedures aseptically and carefully that might break the mucous membrane of newborns and create easy accessibility of pathogenic organisms.

### Strengths and limitations of the study

Having a response rate of 100% and the utilization of a valid and standardized instrument were the strengths of this research. On the other hand, due to the nature of the study design, it could not ascertain a cause-and-effect relationship, and since the study was done on admitted neonates, the results might lack generalizability to the entire population, which was considered a limitation of the study.

## Conclusion

Generally, the current study demonstrates that neonatal sepsis was high, occurring in over half of the neonates admitted to the NICU, which imposes great obstacles to achieving the Sustainable Development Goals (SDGs) call target 3.2 for an end to preventable deaths of newborns by 2030. Moreover, the main predictors of neonatal sepsis were NG tube insertion, resuscitation during birth, and the neonate's mode of delivery. Thus, it is strongly recommended to develop plans to improve the aseptic techniques used by healthcare providers, train healthcare personnel, monitor and assess obstetric and neonatal care, and actively and cooperatively collaborate with cluster health institutions.

## Data Availability

The raw data supporting the conclusions of this article will be made available by the authors, without undue reservation.

## Abbreviation

ANC: Antenatal Care
AOR: Adjusted Odds Ratio
APGAR: Appearance, Pulse, Grimace, Activity, Respiration
CI: Confidence Interval
COR: Crude Odds Ratio
C/S: Cesarean section
EDHS: Ethiopian Demographic Health Survey
EOS: Early-Onset Neonatal sepsis
HUCSH: Hawassa University Comprehensive Specialized Hospital
KMC: Kangaroo Mother Care
LON: Late-Onset Neonatal sepsis
MSAF: Meconium stained amniotic fluid
MDG: Millennium Development Goal
NGT: Nasogastric Tube insertion
NICU: Neonatal Intensive Care Unit
PROM: Premature Rupture of Membrane
SDG: Sustainable Development Goal
SPSS: Statistical Product and Service Solution
SSA: Sub-Saharan Africa
UNICEF: United Nations Children Fund, WHO- World Health Organization
